# Is Your Neighborhood Designed to Support Physical Activity? A Brief Streetscape Audit Tool

**DOI:** 10.5888/pcd12.150098

**Published:** 2015-09-03

**Authors:** James F. Sallis, Kelli L. Cain, Terry L. Conway, Kavita A. Gavand, Rachel A. Millstein, Carrie M. Geremia, Lawrence D. Frank, Brian E. Saelens, Karen Glanz, Abby C. King

**Affiliations:** Author Affiliations: Kelli L. Cain, Terry L. Conway, Kavita A. Gavand, Carrie M. Geremia, University of California, San Diego; Rachel A. Millstein, San Diego State University/University of California, San Diego Joint Doctoral Program in Clinical Psychology, San Diego, California; Lawrence D. Frank, University of British Columbia, Vancouver, British Columbia, Canada; Brian E. Saelens, University of Washington, Seattle, Washington; Karen Glanz, University of Pennsylvania, Philadelphia, Pennsylvania; Abby C. King, Stanford University, Stanford, California.

## Abstract

**Introduction:**

Macro level built environment factors (eg, street connectivity, walkability) are correlated with physical activity. Less studied but more modifiable microscale elements of the environment (eg, crosswalks) may also affect physical activity, but short audit measures of microscale elements are needed to promote wider use. This study evaluated the relation of a 15-item neighborhood environment audit tool with a full version of the tool to assess neighborhood design on physical activity in 4 age groups.

**Methods:**

From the 120-item Microscale Audit of Pedestrian Streetscapes (MAPS) measure of street design, sidewalks, and street crossings, we developed the 15-item version (MAPS-Mini) on the basis of associations with physical activity and attribute modifiability. As a sample of a likely walking route, MAPS-Mini was conducted on a 0.25-mile route from participant residences toward the nearest nonresidential destination for children (n = 758), adolescents (n = 897), younger adults (n = 1,655), and older adults (n = 367). Active transportation and leisure physical activity were measured with age-appropriate surveys, and accelerometers provided objective physical activity measures. Mixed-model regressions were conducted for each MAPS item and a total environment score, adjusted for demographics, participant clustering, and macrolevel walkability.

**Results:**

Total scores of MAPS-Mini and the 120-item MAPS correlated at *r* = .85. Total microscale environment scores were significantly related to active transportation in all age groups. Items related to active transport in 3 age groups were presence of sidewalks, curb cuts, street lights, benches, and buffer between street and sidewalk. The total score was related to leisure physical activity and accelerometer measures only in children.

**Conclusion:**

The MAPS-Mini environment measure is short enough to be practical for use by community groups and planning agencies and is a valid substitute for the full version that is 8 times longer.

## Introduction

To increase physical activity among most Americans who do not meet national guidelines (1), many health organizations have called for built environmental changes to create conditions for safe and convenient physical activity for leisure and transportation purposes (2–7). Built environment attributes are related to physical activity (2,4,5,8), but most evidence is based on studies of “macroscale” features, such as mixed land use, street connectivity, residential density, and proximity to recreation facilities. Evidence about “microscale” features of the built environment, such as pedestrian and bicycle facilities, intersection characteristics, and aesthetics, is limited. Microscale attributes of streetscapes may alter pedestrian and bicyclist experiences related to comfort and safety, but few studies of the relation of audit measures to physical activity could be found (9). A recent study showed microscale features were related to physical activity in 4 age groups (10), supporting the need for increased attention to microscale environments.

A critical advantage of many microscale environmental features is that they are modifiable. It takes less time and money to repair a sidewalk or improve a street crossing than to change a neighborhood’s layout. Several observation-based, or audit, measures of microscale environments have been developed (11), but they are generally too long for use in practice and too complicated to score and interpret results. Many walkability audits are used in practice, but they have not been evaluated. One instrument was developed for use by community groups and was evaluated, but it is long (12), which can be a barrier to use.

The purpose of this study was to develop and evaluate a brief audit instrument that quantifies modifiable attributes of environments, is feasible for use by practitioners, and produces interpretable and valid results. The validated 120-item version of the Microscale Audit of Pedestrian Streetscape (MAPS) instrument (10,13) was the basis for the 15-item version called MAPS-Mini.

## Methods

The original MAPS was adapted from the Analytic Audit Tool (12), as modified by the Healthy Aging Network (14). To maximize the relevance of MAPS to participant physical activity, observations were made on a 0.25-mile route toward the nearest destination from the participant’s residence, assuming this would be a likely walking route. Eligible destinations included commercial centers, parks, and schools. The 4 sections of the original MAPS were route, street segments, crossings, and cul-de-sacs. Route items summarized characteristics for the entire 0.25-mile route and included land use and destinations (which are macroscale variables), speed limit, aesthetics, and transit stops. Segment items were collected on every street block face on the route, including presence and quality of sidewalks, buffers between streets and sidewalks, trees, and setbacks of buildings from streets. Street crossing items were measured at every intersection on the route and included crosswalk markings, width of crossings, and crossing signals. Because routes could contain multiple segments and crossings, these items were averaged for each participant. Cul-de-sac amenities, such as landscaping and basketball hoops, were assessed when available (13).

MAPS-Mini was designed to be short enough to be feasible for use in practice, with modest training of observers. Items were selected by consensus and generally met at least 2 of 3 criteria: 1) measure a modifiable attribute (eg, slope/steepness was deleted because it is not modifiable), 2) correlate with physical activity (primarily active transportation: walking or biking as a form of transportation), and 3) be consistent with practice-based guidelines for activity-supportive environments (15–17). Fourteen items were selected, and an additional item was created to fill a gap in assessment of bicycle facilities, such as bicycle lanes or separated paths. The new item asks, “Is there a designated bike path on the route?” Scoring was based on the extent to which bicyclists were protected from cars: 0 for no identified space for bicyclists (including “sharrows” markings, which do not protect cyclists), 1 for painted bike lanes, and 2 for any physical protection from cars.

Data were collected during 2009 and 2010 by trained and certified raters (13) (forms and training manual at http://sallis.ucsd.edu/measure_maps.html). MAPS items and subscales demonstrated moderate to excellent interobserver reliability (13). Validity was assessed by associations with multiple measures of physical activity, adjusting for macrolevel walkability. Walking or bicycling for transportation and leisure or neighborhood physical activity were measured with age-appropriate surveys, and total physical activity was measured with accelerometers (10). Validity testing demonstrated that 51% of the 43 subscale scores on the original MAPS were significantly associated with walking or bicycling for transport, 22% with leisure or neighborhood physical activity, and 16% with objectively measured moderate to vigorous physical activity (MVPA).

MAPS-Mini was evaluated with the same data sets used to compare the original MAPS with physical activity outcomes (10). The Neighborhood Impact on Kids (NIK) study included 758 children aged 6 to 11 years (31.4% nonwhite) (18,19). The Teen Environment and Neighborhood (TEAN) study included 897 adolescents aged 12 to 16 (33.3% nonwhite) (20). Parents or guardians of NIK and TEAN youth (n = 1,655; 24.2% nonwhite) constituted the adult sample in this study. Senior Neighborhood Quality of Life Study (SNQLS) included 367 older adults aged 66 years or older in Seattle/King County (16.2% nonwhite) (21). Studies were conducted in Seattle/King County, Washington; San Diego County, California; and the Baltimore, Maryland–Washington, DC region. Neighborhoods were selected to vary widely by socioeconomic status and walkability defined by macrolevel environment features and recreation environment; then participants were recruited from selected neighborhoods (10). The studies were approved by San Diego State University and the institutional review boards of other participating investigators.

Each item on MAPS-Mini was scored either 0–1 or 0–1–2. Item scores were averaged across multiple street segments and crossings to compute participant-level scores. The total score was the sum of all computed items for each participant and was intended to represent the cumulative effect of microscale attributes of the built environment. A second scoring method was the “percentage of possible maximum score,” which is more easily interpretable. Because users may want to adapt MAPS-Mini by adding a small number of items specific to their region or interests, using the “percentage of possible maximum score” will allow rough comparability of scores across different versions.

### Physical activity measures

The same physical activity outcomes reported by Cain et al (10) were used in our analyses. Parents (for children in NIK) and adolescents in TEAN reported frequency of walking and bicycling (0 = never to 5 = ≥4 times/wk) to 9 common locations. The mean score was analyzed (22). Parents of child and adolescent participants completed the Global Physical Activity Questionnaire (GPAQ) (23). The active transportation item assessed days walking and biking for transport during a typical week. Older adults completed the Community Healthy Activities Model Program for Seniors (CHAMPS) (24). Participants reported times per week they usually walked or biked for errands (biking added for SNQLS) in an open-ended format.

Parents (for children in NIK) and adolescents in TEAN reported the frequency with which they were physically active near home in 5 places such as nearby streets, sidewalks, and cul-de-sacs (0 = never to 5 = ≥4 times a week) (22). Parents reported minutes per typical day they spent in leisure physical activity on the GPAQ. For older adults, a CHAMPS item about time walking for leisure was used.

Objective physical activity was measured using the Actigraph (Actigraph, LLC) accelerometer (models 7164/71256 for adolescents/older adults; GT1M/GT3X with normal filter for children/adolescents). Parents in NIK and TEAN did not wear accelerometers. Accelerometers collected data in 30-second (children and adolescents) or 60-second intervals (older adults). Participants wore the accelerometer on the waist for 7 days during waking hours (except when swimming or bathing). After their return, Actigraphs were downloaded and screened for completeness using MeterPlus versions 4.0 through 4.3 (www.meterplussoftware.com). A valid day had at least 10 valid hours of wear time. Nonwearing time was 20, 30, or 45 minutes of consecutive zero counts for children, adolescents, and older adults, respectively (25). Participants with inadequate wear time were asked to re-wear the device.

For adolescents, data were scored using Freedson youth age-specific cut points with a 4-MET moderate intensity cut point of 4 metabolic equivalents of task (multiples of rest; METs) (26). Average daily minutes of MVPA only during nonschool hours were computed (3 PM–11 PM on weekdays; all hours on weekends). For children, MVPA in the neighborhood was calculated on the basis of parent report of times in neighborhood locations that were temporally linked to accelerometer data (27). Average daily minutes of MVPA in the neighborhood were calculated using the Freedson youth age-specific 3-MET cut point (26). For older adults, average daily minutes in MVPA were computed using the Freedson adult 3-MET cut point (28).

Demographic covariates assessed by survey were participant age, sex, education (parent education for children and adolescents), and race/ethnicity. Education was dichotomized as college degree or higher versus less, and race/ethnicity was dichotomized into white/non-Hispanic or nonwhite (including Hispanic). Older adults reported on lower-extremity mobility impairment measured with the 11-item subscale of the Late-Life Function and Disability Instrument (29). Macrolevel neighborhood walkability was assessed by using an index created with geographic information systems: net residential density, street connectivity, land use mix, and retail floor area ratio (18,30). Neighborhoods were categorized as higher or lower on the walkability index in their respective regions.

Mixed linear regression analyses assessed the relation of each MAPS-Mini item with physical activity outcomes for each age group, adjusting for all covariates, including macrolevel walkability, as fixed effects and participant clustering in census block groups (per recruitment procedures) as a random effect. We present *t* statistics from the adjusted mixed models (Tables 1, 2, and 3) instead of β estimates and confidence intervals because *t* statistics (and significance levels) provide a common indicator for comparing relative magnitudes of association across MAPS-Mini scores.

**Table 1:** Mixed Regression Results of Relationship Between MAPS-Mini Scores and Walking and Biking for Transport, 3 US Cities, 2009–2010

Variables	Children^a^		Adolescents^a^		Adults^a^		Older Adults^a^	
	*t* Value^b^	*P* Value^b^	*t* Value^b^	*P* Value^b^	*t* Value^b^	*P* Value^b^	*t* Value^b^	*P* Value^b^
**Destinations and land use**								
Public park	0.53	.60	1.06	.29	.78	.44	−1.51	.13
**Streetscape characteristics**								
Transit stops	1.80	.07	1.87	.06	3.77	<.001	0.23	.82
Street lights	2.66	.008	2.94	.003	2.98	.003	0.11	.91
Benches	2.63	.009	0.23	.82	2.76	.006	2.14	.03
**Aesthetics and social characteristics^c^**								
Building maintenance	0.89	.37	−1.45	.15	−1.33	.18	−2.28	.02
Absence of graffiti	0.77	.44	−0.38	.70	−2.36	.02	0.14	.89
**Crossings/intersections**								
Crosswalk	1.75	.08	0.40	.69	1.30	.20	2.15	.03
Curb cuts	3.57	<.001	−0.06	.95	2.39	.02	2.38	.02
Crossing signal	−0.76	.45	0.19	.85	0.21	.84	2.63	.009
**Street segments**								
Commercial	1.52	.13	1.83	.07	4.44	<.001	^ — ^ d	^ — ^ d
Sidewalk	4.82	<.001	1.06	.29	3.05	.002	2.17	.03
Sidewalk buffer	3.25	.001	2.04	.04	4.72	<.001	1.73	.09
Trees and overhead coverage	0.33	.75	1.50	.14	1.98	.05	−0.05	.96
Absence of trip hazards	3.34	.001	0.23	.82	2.03	.04	1.72	.09
**Total score**	5.22	<.001	2.47	.01	5.59	<.001	2.15	.03

Abbreviation: MAPS, Microscale Audit of Pedestrian Streetscapes.

a Analyses adjusted for age, sex, education, race/ethnicity, geographic information services (GIS)-defined walkability (high/low), physical functioning for older adults, and clustering of participants within block groups.

b *P* values and *t* values calculated from mixed regressions.

c Not included in total score.

d Data not available.

**Table 2:** Mixed-Regression Results for Relationship Between MAPS-Mini Scores and Leisure and Neighborhood Physical Activity, 3 US Cities, 2009–2010

Variables	Children^a^ (in Neighborhood)		Adolescents^a^ (in Neighborhood)		Adults^a^		Older Adults^a^	
	*t* Value^b^	*P* Value^b^	*t* Value^b^	*P* Value^b^	*t* Value^b^	*P* Value^b^	*t* Value^b^	*P* Value^b^
**Destinations and land use**								
Public park	1.78	.08	0.11	.91	0.24	.81	−0.21	.83
**Streetscape characteristics**								
Transit stops	−2.30	.02	0.03	.98	−0.22	.83	−0.08	.94
Street lights	0.57	.57	−0.86	.39	0.23	.82	−0.23	.82
Benches	1.04	.30	−2.84	.005	1.61	.11	0.93	.35
**Aesthetics and social characteristics**								
Building maintenance	2.96	.003	−2.36	.02	3.65	<.001	−0.25	.80
Absence of graffiti	2.49	.01	1.32	.19	3.57	<.001	−0.30	.76
**Crossings/intersections**								
Crosswalk	−0.25	.81	−1.86	.06	0.19	.85	0.89	.37
Curb cuts	2.76	.006	−1.72	.09	−0.62	.54	0.84	.40
Crossing signal	−1.71	.09	−2.22	.03	0.67	.51	1.32	.19
**Street segments**								
Commercial	−3.13	.002	−1.59	.11	−1.07	.29	—c	—c
Sidewalk	2.15	.03	−0.79	.43	−1.36	.17	1.18	.24
Sidewalk buffer	−0.09	.93	0.33	.74	−1.52	.13	−0.45	.65
Trees and overhead coverage	2.35	.02	0.13	.90	−0.40	.69	−0.26	.80
Absence of trip hazards	2.10	.04	0.09	.93	−1.56	.12	1.24	.22
**Total score**	2.46	.01	−1.69	.09	0.32	.75	0.80	.43

Abbreviations: GIS, geographic information systems; MAPS, Microscale Audit of Pedestrian Streetscapes.

a Analyses adjusted for age, sex, education, race, GIS-defined walkability (high/low), physical functioning for older adults, and clustering of participants within block groups.

b *P* values and *t* values in table are calculated from mixed regressions.

c Data not available.

**Table 3:** Mixed-Regression Results for Relationship Between MAPS-Mini Scores and Accelerometer-Derived Total MVPA Minutes per Day, 3 US Cities, 2009–2010

Variables	Children^a^ (in neighborhood)		Children^a^ (nonschool time)		Adolescents^a^ (in neighborhood)		Older Adults^a^	
	*t* Value^b^	*P* Value^b^	*t* Value^b^	*P* Value^b^	*t* Value^b^	*P* Value^b^	*t* Value^b^	*P* Value^b^
**Destinations and land use**								
Public park	1.28	.20	−0.32	.75	1.59	.11	−0.65	.52
**Streetscape characteristics**								
Transit stops	1.23	.22	0.37	.71	0.72	.47	0.50	.62
Street lights	1.02	.31	−1.78	.08	−0.25	.81	−0.14	.89
Benches	0.13	.90	1.83	.07	−0.59	.56	0.77	.44
**Aesthetics and social characteristics**								
Building maintenance	0.92	.36	0.99	.32	−0.69	.49	0.55	.58
Absence of graffiti	1.76	.08	1.30	.20	0.66	.51	0.75	.46
**Crossings/intersections**								
Crosswalk	−1.09	.28	−0.89	.37	0.52	.61	0.76	.45
Curb cuts	2.93	.004	0.80	.42	0.86	.39	1.10	.27
Crossing signal	−1.35	.18	−1.90	.06	0.74	.46	1.21	.23
**Street segments**								
Commercial	0.06	.95	−1.62	.11	−0.54	.59	—c	—c
Sidewalk	1.97	.05	−0.56	.57	1.05	.29	1.76	.08
Sidewalk buffer	1.09	.28	−0.24	.81	0.78	.44	−0.03	.97
Trees and overhead coverage	0.88	.38	−0.57	.57	0.17	.86	0.74	.46
Absence of trip hazards	1.19	.24	−0.49	.62	0.67	.51	1.04	.30
**Total score**	2.69	.007	−0.28	.78	1.19	.23	1.48	.14

Abbreviations: MAPS, Microscale Audit of Pedestrian Streetscapes; MVPA, moderate to vigorous physical activity.

a Analyses adjusted for age, sex, education, race, GIS-defined walkability (high/low), physical functioning for older adults, and clustering of participants within block groups.

b *P* values and *t* values are calculated from mixed regressions.

c Data not available.

## Results

The median of the MAPS-Mini total score was 37%, with nearly all the scores between 5% and 72%. The correlation of the total scores for the original 120-item MAPS and MAPS-Mini, including all participants, was *r* = 0.85.

There were 28 significant associations (*P ≤ .*05) with MAPS-Mini items and walking or bicycling for transport across all age groups (46.7% associations with MAPS-Mini) (Table 1). Street lights, benches, curb cuts, the presence of a sidewalk, and buffers between streets and sidewalks were related to active transport in 3 of 4 age groups. Aesthetics and social characteristics were largely unrelated to active transport. Children and younger adults had the highest number of significant items. Crossings and intersections were particularly important for older adults, as all 3 crossings items were related to active transport. Only 2 individual items (street lights and sidewalk buffer) were significant for adolescents. The total score was significantly related to walking and biking for transport in all age groups.

There were 14 significant associations with MAPS-Mini items and leisure or neighborhood physical activity across all age groups (23.3% of associations with MAPS-Mini) (Table 2). Aesthetics and social characteristics were positively related to leisure physical activity in children and younger adults, and streetscape characteristics (transit stops and benches) were negatively related in children and adolescents. Children had the highest number of significant items, including curb cuts, presence of a sidewalk, trees and overhead coverage, and absence of trip hazards. Several significant associations were found with adolescents’ reported neighborhood physical activity, but in the unexpected direction. That is, MAPS items were negatively correlated with adolescents’ neighborhood physical activity. There were no significant relationships in older adults. The total score was related to neighborhood physical activity in children only.

There were only 3 significant associations with MAPS-Mini items and objectively measured MVPA (5% of associations with MAPS-Mini) (Table 3). Presence of a sidewalk, curb cuts, and the total score were related to “in neighborhood” MVPA in children.

**Figure 1:**
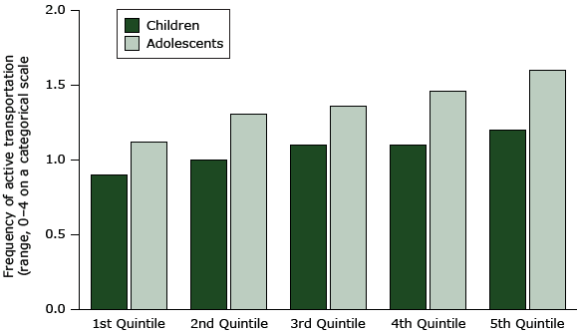
Association of active transport with MAPS-Mini scores (percentage of total possible) ranked in quintiles from the poorest (lowest quintile) to the best (highest quintile) activity supportive microscale attributes of the built environment in the 2 younger age groups. Quintiles for children ranged from 13.3% to 54.0% and quintiles for adolescents, 15.7% to 61.9%.

**Figure 2:**
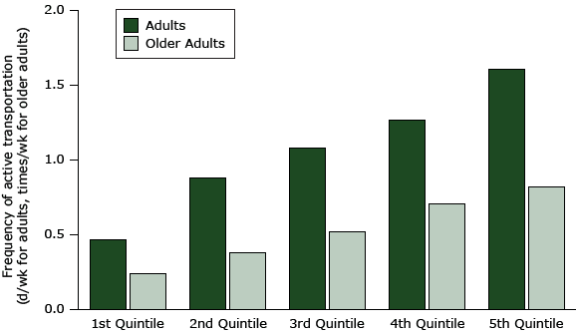
Association of active transport with MAPS-Mini scores (percentage of total possible) ranked in quintiles from the poorest (lowest quintile) to the best (highest quintile) activity supportive microscale attributes of the built environment in the 2 older age groups. Quintiles for younger adults ranged from 14.6% to 59.2%; for older adults, 14.4% to 64.0%. For a complete comparison of quintile total scores with age group activity scores, see Appendix.

Figures 1 and 2 show the linear relation of the MAPS-Mini score (percentage of total possible) and reported active transport in all age groups. Older adults in the lowest quintile for MAPS-Mini scores walked for transport an average of 0.2 times per week, while those in the highest quintile walked an average of 0.8 times per week, a difference of 0.6 times per week. The respective difference between the lowest and highest quintiles in younger adults was 1.1 days per week. For a complete comparison of quintile total scores with age group activity scores, see Appendix. Although the relationships of MAPS-Mini total scores and active transport for children and adolescents were linear, as shown in the figures, the magnitude of effect was difficult to interpret because of the categorical response scale (never to ≥4 times per week).

**Table:** 

Quintile	Children	Adolescents
	Active Transport (Category Score Range, 0-4)	Active Transport (Category Score Range, 0-4)
1st	0.9	1.1
2nd	1.0	1.3
3rd	1.1	1.4
4th	1.1	1.5
5th	1.2	1.6

**Table:** 

Quintile	Younger Adults	Older Adults
	Active Transport (d/wk)	Active Transport (times/wk)
1st	0.5	0.2
2nd	0.9	0.4
3rd	1.1	0.5
4th	1.3	0.7
5th	1.6	0.8

## Discussion

The 15-item MAPS-Mini total microscale environment score was significantly related to walking and bicycling for transportation in all 4 age groups. Improving microscale features of the built environment such as those on MAPS-Mini has the potential to facilitate more active transportation, independent of the walkability of the neighborhood. A high correlation between MAPS-Mini and the full MAPS indicates that the 15 items in MAPS-Mini provide an efficient and useful measure of the physical activity supportiveness of neighborhood environments that is feasible for use by nonresearchers.

Five items were significantly related to active transportation in 3 age groups: sidewalk presence, curb cuts, street lights, benches, and buffer between street and sidewalk. These attributes could be particularly important for improving the experience of pedestrians and bicyclists, or they could be indicators of a broader pattern of activity-supportive design features. For example, it could be that streets with curb cuts and benches are also designed to benefit pedestrians in multiple ways, such as increasing the safety of intersections, slowing traffic speeds, or providing aesthetic elements such as colorful buildings. Sidewalks may be the most basic attribute for supporting pedestrian activity. Curb cuts improve access for older adults, people with disabilities, and parents with baby strollers. Benches may be an unexpected correlate of physical activity, but their presence signals consideration for pedestrians, and they may be important for children or older adults who need a place to rest during walks. Street lights are needed for nighttime activity and to increase feelings of security. Separating pedestrians from traffic with a planting strip or parked cars improves safety and pedestrian comfort. Thus, the items with the most consistent associations with active transport appear to serve a variety of functions.

The figures show that the associations of MAPS-Mini scores with active transport are linear and positive for all age groups. The implication is that making one improvement to streetscape environments would probably have a small effect, but making several improvements could have cumulatively large effects on walking and bicycling for transportation. We illustrated the strength of associations (ie, effect sizes) by comparing the active transportation scores for the lowest versus the highest quintiles of MAPS-Mini scores. The differences were 33% for children, 43% for adolescents, 243% for adults, and 242% for older adults. The effect sizes may be lower for children in part because of the smaller range (0–4) of the categorical responses and in part because of higher rates of active transport overall. The reported frequency of walking and cycling for transport was very low for adults and older adults, so modest differences produced large percentage changes. These differences indicate that the potential for increasing active transportation by making microscale environmental changes is substantial. Findings for adults and older adults suggested that large improvements in MAPS-Mini scores might lead to an almost 250% increase in walking for transportation, but from a low baseline of much less than 1 time per week. An increase of 1.1 times per week walking or bicycling would be a big change for many adults.

Streetscape environments appear to be less important for leisure physical activity than for active transportation. The 2 aesthetic items of building maintenance and absence of graffiti had inconsistent associations with leisure physical activity. The total score was significantly related only to children’s leisure activity (in the neighborhood), with 6 items significant in the expected positive direction and 2 items (transit stops, commercial segments) significant in the unexpected negative direction. Busy commercial areas with multiple transit stops may not be perceived by parents as safe places for children to play.

There was little indication that microscale environmental attributes were related to total MVPA measured by accelerometer. This could be because people are physically active in various settings outside the home neighborhood, including workplace, school, work and school neighborhoods, and recreation facilities. Children’s total MVPA was significantly related to their MAPS-Mini total scores, although only 2 items were significant (sidewalks, curb cuts). This finding indicates that total scores, representing a more complete measure of a physical activity-supportive pattern of microscale attributes, can have stronger associations with outcomes than individual items.

MAPS-Mini offers a useful assessment of environmental support for active transport. Only one participant’s route scored above 80% of the maximum score, and half the routes scored under 37% of the maximum score, indicating substantial room for improvement.

Study strengths included systematic development of a brief streetscape environment audit instrument, validation with 4 age groups from 3 regions of the United States, multiple physical activity measures used in validation analyses, and statistical adjustment for macrolevel walkability. One weakness was that self-reported physical activity measures were not comparable across age groups, in part because they were selected to be age-appropriate. Another limitation was the inadequate assessment of bicycle facilities, such as separated paths, and the new item needs to be evaluated. MAPS-Mini includes some macroscale items (commercial land use, public parks) along with microscale assessments, but the macroscale items may improve the usefulness of the measure for overall physical activity supportiveness. Because the current validation was conducted with a subset of items from the original version, the validity of MAPS-Mini should be tested as a stand-alone instrument. It would be useful to evaluate the feasibility with which practitioners and community members can reliably conduct MAPS-Mini observations with brief training. Demonstrating that nonresearchers without a specialized educational background can use MAPS-Mini would allow this measure to empower community members to be involved in city planning processes and provide city planners with more precise measures of streetscapes.

MAPS-Mini was useful as a measure of streetscape quality that was mainly related to active transport in all 4 age groups. MAPS-Mini total scores were linearly related to active transport in all age groups, suggesting that multiple environmental attributes supportive of activity need to be provided or improved to have a large effect on walking and bicycling. Thus, the total score seems to be the best indicator of activity supportiveness, and no single attribute was dominant in encouraging active transport. Microscale environment attributes may be most important for children, because children were the only group for which MAPS-Mini scores were significantly related to all 3 outcomes.

Although the 15-item MAPS-Mini was highly correlated with the 120-item version, there were some reductions in effect sizes. An important loss with MAPS-Mini was in the ability to examine subscales, because the full version had more than 40 subscale scores that could be useful for informing both research and practice. Thus, the original and MAPS-Mini versions provide options for streetscape assessments that allow researchers and practitioners to choose the version that better suits their preferred level of detail in the data and available resources. We recommend the MAPS-Mini as a feasible, valid, and evidence-based measure that can be used in practice to identify community environment strengths and weaknesses as an aid to planning and evaluating improvements to streetscapes.

## Appendix

This file is available for download as a Microsoft Word document.
