# Supplementary material for: Is Your Neighborhood Designed to Support Physical Activity? A Brief Streetscape Audit Tool
Source: Prev Chronic Dis. 2015 Sep 3;12:E141. doi: 10.5888/pcd12.150098 (PMC4561538; doi:10.5888/pcd12.150098)
Supplement: Supplementary file 1 [file 15_0098_Appendix.docx]

Appendix.

This file is available for download as a Microsoft Word document [DOC – XXKB].

**15-Item Streetscape Audit for Physical Activity-Supportiveness of Environments: MAPS-Mini**

| **Quintile** | **Children** | | **Adolescents** | | **Adults** | | **Older Adults** | |
| --- | --- | --- | --- | --- | --- | --- | --- | --- |
|  | **Percent of Total Score^a^** | **Active Transport (no. of times/wk)** | **Percent of Total Score^a^** | **Active Transport (no. of times/wk)** | **Percent of Total Score^a^** | **Active Transport (d/wk)** | **Percent of Total Score^a^** | **Active Transport (d/wk)** |
| 1st | 13.3 | 0.9 | 15.7 | 1.12 | 14.6 | 0.47 | 14.4% | 0.24 |
| 2nd | 29.2 | 1.0 | 32.8 | 1.31 | 31.4 | 0.88 | 27.7% | 0.38 |
| 3rd | 36.8 | 1.1 | 42.0 | 1.36 | 39.3 | 1.08 | 38.1% | 0.52 |
| 4th | 42.7 | 1.1 | 49.4 | 1.46 | 46.3 | 1.27 | 50.2% | 0.71 |
| 5th | 54.0 | 1.2 | 61.9 | 1.60 | 59.2 | 1.61 | 64.0% | 0.82 |

MAPS-Mini scores (percentage of total possible score) were used to group participant routes by quintiles based on having microscale attributes of the built environment that were least supportive (lowest quintile) to most supportive (highest quintile) of physical activity. Positive linear associations between MAPS-Mini scores and active transportation were found in each age group. Compared with the lowest quintile, participants in the highest quintile reported more frequent active transportation ranging from 33% higher for children, 43% for adolescents, 243% for adults, and 242% higher for older adults. One interpretation of the figure is that there is no basis for a minimum or maximum recommendation for an activity-supportive microscale environment; each increment in MAPS-Mini scores is associated with more frequent walking for transportation. MAPS-Mini items can be considered a rough guide to creating environments that provide positive experiences for pedestrians. Abbreviation: MAPS, Microscale Audit of Pedestrian Streetscapes.
